# Supplementary material for: Impact of assessment and intervention by a health and social care professional team in the emergency department on the quality, safety, and clinical effectiveness of care for older adults: A randomised controlled trial
Source: PLoS Med. 2021 Jul 28;18(7):e1003711. doi: 10.1371/journal.pmed.1003711 (PMC8318294; doi:10.1371/journal.pmed.1003711)
Supplement: S1 Intervention Characteristics — (DOCX) [file pmed.1003711.s002.docx]

**Impact of assessment and intervention by a Health and Social Care Professional team in the emergency department on the quality, safety, and clinical effectiveness of care for older adults: a randomised controlled trial**

**S1 Intervention characteristics**

| **HSCP assessment and intervention** |  |
| --- | --- |
| Subjective assessment | - Detailed information gathering from patient regarding nature of presenting complaint - Past medical history and impact of condition(s) on functional status and psychosocial well-being and QoL - Healthcare utilisation and formal/informal support network in place - Determination of will and preference re: expectation of ED care and disposition plan - Detailed history of baseline functional and mobility status; multifactorial falls history; use of telecommunications in the home |
| Objective assessment | Observation   - Levels of alertness and/or pain - Signs of deformity   Cognition   - Delirium screening (completion of 4AT) - Assessment of functional cognition and administration of formal cognitive screens, as indicated e.g. MOCA V8   Physical   - Palpation of affected area (if limb injury) and assessment of pain - Assessment of active and passive range of motion of all limbs - Assessment of strength (manual muscle testing), sensation, coordination, reflexes. More detailed neurological assessment completed, if indicated   Function-focused measures   - Assessment of functional transfers and mobility. Stairs assessment completed, as indicated - Balance: Timed Up and Go, Single Leg Stand, Berg Balance - Activities of daily living (ADL): assessment of personal ADLS within ED environment e.g. ability to complete e.g. toileting and continence management   Psychosocial   - Screening for safeguarding of vulnerable adults |
| Evaluation | Interdisciplinary analysis of all subjective and objective findings and coordination of an intervention plan. Interventions typically employed included;   - Prescription of mobility aids and enabling equipment to compensate for residual functional deficits - Education re: activity modification and self management strategies - Provision and education on Home Exercise Programmes - Family and carer education, as indicated - Shared decision making with patient regarding care planning e.g. application for home supports and onward referrals to primary care services |
| Plan | Discussion with EM team and shared decision-making regarding proposed interdisciplinary discharge plan |
