# Supplementary material for: Impact of assessment and intervention by a health and social care professional team in the emergency department on the quality, safety, and clinical effectiveness of care for older adults: A randomised controlled trial
Source: PLoS Med. 2021 Jul 28;18(7):e1003711. doi: 10.1371/journal.pmed.1003711 (PMC8318294; doi:10.1371/journal.pmed.1003711)
Supplement: S1 Sensitivity Analyses — (DOCX) [file pmed.1003711.s003.docx]

**Impact of assessment and intervention by a Health and Social Care Professional team in the emergency department on the quality, safety, and clinical effectiveness of care for older adults: a randomised controlled trial**

**S1 Sensitivity Analyses on outcomes ED length of stay and hospital admissions.**

Analyses controlled for: triage category, patient’s age, patient’s baseline ISAR score, nutritional status, and clinical frailty score.

| **Sensitivity Analysis Table 1 \| ED length of stay – Linear Regression** | | | | | | |
| --- | --- | --- | --- | --- | --- | --- |
|  | **Model 1** | | | **Model 2** | | |
| **Measure** | **b** | **p-value** | **95% CI** | **b** | **p-value** | **95% CI** |
| Arm (Ref: Control) | -6.61 | < 0.001 | -10.04: -3.17 | -5.72 | 0.001 | -9.12: -2.33 |
| Triage category (Ref: MTS 2) |  |  |  |  |  |  |
| MTS 3 |  |  |  | -6.66 | 0.019 | -12.21 ; -1.12 |
| MTS 4 |  |  |  | -11.85 | 0.007 | -20.43 : -3.28 |
| Age |  |  |  | 0.15 | 0.23 | -0.09 : 0.41 |
| ISAR score (Ref: <2) |  |  |  |  |  |  |
| >=2 |  |  |  | 1.75 | 0.46 | -2.97 : 6.48 |
| Nutritional status (Ref: 1. Malnourished) |  |  |  |  |  |  |
| At risk of malnutrition |  |  |  | 5.38 | 0.13 | -1.54 : 12.31 |
| Normal status |  |  |  | -0.89 | 0.79 | -7.73 : 5.93 |
| Clinical Frailty Score |  |  |  | 1.27 | 0.09 | -0.23 : 2.78 |
| F | 14.30 | < 0.001 |  | 6.40 | < 0.001 |  |
| Adjusted R^2^ | 0.03 |  |  | 0.11 |  |  |
| **Notes**. b = unstandardised regression coefficient, CI = Confidence Interval, ED= Emergency Department, F = Anova F-ratio, R^2^ = coefficient of determination, Ref = Reference category. | | | | | | |

| **Sensitivity Analysis Table 2 \| Hospital admissions – Logistic Regression** | | | | | | |
| --- | --- | --- | --- | --- | --- | --- |
|  | **Model 1** | | | **Model 2** | | |
| **Measure** | **OR** | **p-value** | **95% CI** | **OR** | **p-value** | **95% CI** |
| Arm (Ref: Control) | 0.18 | < 0.001 | 0.12 : 0.31 | 0.21 | < 0.001 | 0.12: 0.34 |
| Triage category (Ref: MTS 2) |  |  |  |  |  |  |
| MTS 3 |  |  |  | 1.13 | 0.78 | 0.48 : 2.64 |
| MTS 4 |  |  |  | 0.32 | 0.15 | 0.06 : 1.53 |
| Age |  |  |  | 1.03 | 0.16 | 0.99 : 1.07 |
| ISAR score (Ref: <2) |  |  |  |  |  |  |
| >=2 |  |  |  | 1.61 | 0.21 | 0.77 : 3.35 |
| Nutritional status (Ref: 1. Malnourished) |  |  |  |  |  |  |
| At risk of malnutrition |  |  |  | 1.37 | 0.52 | 0.52 : 3.63 |
| Normal status |  |  |  | 0.58 | 0.26 | 0.23 : 1.51 |
| Clinical Frailty Score |  |  |  | 1.16 | 0.18 | 0.93 : 1.51 |
| Likelihood ratio chi^2^ | 52.06 | < 0.001 |  | 86.31 | < 0.001 |  |
| Pseudo R^2^ | 0.11 |  |  | 0.18 |  |  |
| **Notes**. CI = Confidence Interval, ED= Emergency Department, OR = Odds Ratio, R^2^ = coefficient of determination, Ref = Reference category. | | | | | | |
