# Supplementary material for: Impact of assessment and intervention by a health and social care professional team in the emergency department on the quality, safety, and clinical effectiveness of care for older adults: A randomised controlled trial
Source: PLoS Med. 2021 Jul 28;18(7):e1003711. doi: 10.1371/journal.pmed.1003711 (PMC8318294; doi:10.1371/journal.pmed.1003711)
Supplement: Attachment — Submitted filename: revisione plos medicine16_12[16876].docx [file pmed.1003711.s004.docx]

Randomised controlled trial of the impact of a Health and Social Care Professional team in the emergency department on the quality, safety, and clinical effectiveness of care for older adults.

Thank you for the opportunity to review this interesting paper.

The paper provides results from a randomized trial aiming to assess the quality, safety, and clinical effectiveness of an intervention based on an early assessment and intervention by a dedicated Health and Social Care Professionals (HSCPs) comprising a senior medical social worker, senior occupational therapist and senior physiotherapist, to promote a safe and timely discharge for older adults in ED.

The study is very nice and explore a topic which is important for older adults.

Older adults in the Emergency Department are at risk of multiple adverse outcomes. The ED is an unsuitable setting for their care, given the scarcity of staff, the long boarding time, the impossibility of ensuring adequate nursing care (i.e. mobilisation, hydration).

The ED Lenght of stay is associated with a higher risk of delirium, an increased rate of in-hospital admissions and in-hospital lkenght of stay , and an increased mortality rate, also after discharge. Treducing A reduction in the length of stay in ED contributes to reduce the overcrowding , which is one of the main factors that affect the quality of care in this setting.

The authors found a significative reduction in length of stay and in the rate of in-hospital admissions. The intervention is effective to improve function, mobility and self care after 30 days and six months after discharge and the satisfaction for the ED admission is higher in intervention group than in control group.

The aim of the study is clear, the title is relevant and informative, the references are recent and relevant. The authors published recently a review about the topic. The paper is well written and clear.

The introduction explains clearly what is already know about this topic and the introduction provide a strong rationale for the study. The research question is clearly outlined and the study is innovative due to the lack of RCT in this topic.

Despite this, I have some comments about the paper.

**1)	The main outcome of the study is the length of stay in Emergency Department.**

Please, specify what did the authors mean for length of stay: time between arrival and discharge? Time between clinical examination and discharge? Time between arrival at triage and clinical examination?

The baseline assessment is comprehensive and in the paper is not specified the time needed to complete it. The time spent for the evaluation is included in the length of stay?

‘…’Patients who presented with a MTS score of very urgent and were categorised as chest pain, abdominal pain, shortness of breath or headache were invited to take part following consultation with the EM doctor and completion of a medical and diagnostic work-up.’’ What is the effect of the intervention on the length of stay in patients who are enrolled after the completion of the medical and diagnostic investigations?

It is well established that the reasons for a prolonged length of stay in ED are several: waiting time for diagnostic exams, waiting for consultants, waiting time for boarding patients…

*… interventions prescribed by the HSCP … included prescription of mobility aids and enabling ADL equipment, provision of home exercise programmes, education of self-management strategies and onward referral to alternative care pathways, as deemed appropriate.*

Please, provide more informations about the intervention and explain how the intervention can reduce the time spent in ED.

**2)	Patients with different clinical conditions were enrolled in the study.**

Patients with chest pain, abdominal pain, dyspnea and headache have different needs in ED than patients with minor complaints. For example, a patient with chest pain often requires several blood exams, taking more time than other complaints. So, patients who are admitted in ED with chest pain or abdominal pain or shortness of breath are expected to have to have a much longer waiting time than others.

‘*’Participants in the intervention group (n=176) experienced a significantly shorter ED stay when compared to the control group (n=177) (6.4 vs. 12.1 median hours, p < 0.001) and had a significantly lower rate of hospital admissions from the ED (19.3% vs. 55.9%, p < 0.001). ‘’*

Is it possible that this difference is related to the reason for admission in ED? How the intervention can reduce the rate of in-hospital admission? The decision to admit a patient in hospital is related to the clinical condition and, sometimes, to a social problem. The decision to admit or discharge a patient is a medical disposition and I’m not sure that the team can influence the decision to discharge a patient. It is not clear how the intervention can influence a clinical decision.

The difference found in the rate of hospital admission is very high, but probably the efficacy of the intervention in the rate of admission is different for patient whit minor complaints than in other.

The reduction in length of stay and in the rate of hospital admission are probably different among patients admitted with 2 or 4-5 MTS score.

In the limits section the authors stated

*We focused on a specific cohort of ‘lower urgency’ patients with a range of index complaints that were amenable to HSCP intervention.*

However, the inclusion criteria state that patients with an acuity level 2 were enrolled after clinical examination. I’m not convinced that chest pain or abdominal pain can be considered fully amenable to HSCP intervention.

**3) inclusion and exclusion criteria**

The inclusion and exclusion criteria are not stated clearly. In the method section *Prospective patients who had a Manchester Triage System score (MTS) of urgent, standard or non-urgent and who were categorized at triage with limb problems, falls, unwell adult, back pain, urinary problems or ear/facial problems were invited to take part.*

But in table 2 there are listed 65 patients admitted for other conditions; major trauma, gastrointestinal bleeding, collapsed adult; gastrointestinal bleeds, head injury... Please, specify the inclusion and exclusion criteria.

Medically unstable: please, specify what this means (ie blood pressure under 90 mmHg, heart rate, news score…). Change with ‘’immediate’’ if the authors mean ''1'' code in MTS scale, or state if it refers to patients unstable also after the clinical examination and treatment. Patients admitted with very urgent condition are clinically unstable, but they were enrolled after medical examination. Please, clarify these inclusion and exclusion criteria.

**Minor comments:**

Please, provide a p-value for characteristics of the sample showed in table 1

Table 2: A category is missed in table 1 for MTS score (2:very urgent-3:urgent-4standard?). Patient non urgent?

It could be interesting to assess the delta in the value of Barthel index and EuroQol in the same patient. An improvement after discharge can be reasonable due to the resolution of the acute clinical condition.

In the sample size it was estimated a 40% decrease in ED length of stay in the intervention group. The authors hypothesized a high reduction in the length of stay, despite previous studies are discordant. For example, Arendts and colleagues reported no significant differences in terms of hospital length of stay after admission from the ED between ED-based CCT assessment of older patients as compared to routine medical assessment (median length of stay: 88 hours in intervention vs. 87 in control, IRR = 0.97, p = 0.32).

How did the researchers calculate the MMSE score?

Follow-up: *The research nurse collected data on objective measures (e.g., ED length of stay, admission rates, ED re-visits, unscheduled hospital admissions) using the hospital routine databases, …* Is it possible that ED visits or hospitalizations in other hospital than the hospital of the study have been lost? Is the hospital routine database inclusive of other hospitals?

Did the telephone call for the follow-up included a reminder of indications provided at discharge?

Please, describe the setting (ie number of patients admitted per year, rate of patients whit low severity at triage per year) and the place where the evaluations were performed (a dedicated room?)
