# Supplementary material for: Impact of assessment and intervention by a health and social care professional team in the emergency department on the quality, safety, and clinical effectiveness of care for older adults: A randomised controlled trial
Source: PLoS Med. 2021 Jul 28;18(7):e1003711. doi: 10.1371/journal.pmed.1003711 (PMC8318294; doi:10.1371/journal.pmed.1003711)
Supplement: Attachment — Submitted filename: PMEDICINE-D-20-05367R1_ResponseToReviewers.docx [file pmed.1003711.s005.docx]

**Subject**: Submission of Revised Research Article (R1) titled “*Randomised controlled trial of the impact of a Health and Social Care Professional team in the emergency department on the quality, safety, and clinical effectiveness of care for older adults*”.

Response to Reviewers

Requests from the editors:

| **Editor Comment** | **Response** |
| --- | --- |
| E1: Please ensure that the data statement complies with PLOS' data policy (<https://journals.plos.org/plosmedicine/s/data-availability>), noting that study authors cannot be listed as points of contact for readers interested in inquiring about access to study data. | We have uploaded the dataset together with codebook to the OSF platform. This is available at the public link <https://osf.io/5wpjm/>.  This information has been included in the paper metadata information in the submission system |
| E2: Please adapt the title so that the study descriptor appears after a colon (i.e., "...: a randomized controlled trial"). | We have adapted the title as requested. |
| E3: Please restructure your abstract into a three-part structure preferred at PLOS Medicine. The final sentence of the "Methods and Findings" subsection should begin "Study limitations include ..." or similar and quote 2-3 of the study's main limitations. | We have restructured the abstracts as requested and added the study limitations as indicated. |
| E4: Please quote summary demographic characteristics for study participants in the abstract. | We have included this information in the new Methods and Findings subsection of the abstract. |
| E5: After the abstract, we will need to ask you to add a new and accessible "author summary" section in non-identical prose. You may find it helpful to consult one or two recent research papers in PLOS Medicine to get a sense of the preferred style. | We have now added an author summary after the abstract, pp. 4-5 |
| E6: Please indicate in the methods section if consent was "informed". | We have now clarified that consent was informed at p.9 |
| E7: Please substitute "sex" for "gender" where appropriate. | We have checked the manuscript and can confirm that “sex” does not appear in the text. |
| E8: Throughout the text, please format reference call-outs as follows: "... poorer health outcomes [4,5].". | We have updated the reference style to the PLOS Medicine format. |
| E9: Please remove the information on funding, competing interests and data sharing from the end of the main text. In the event of publication, this information will appear in the article metadata via entries in the submission form. | We have removed the information on funding, competing interests and data sharing from the article statements as required. |
| E10: Please ensure that all reference citations contain full access information (e.g., reference 4). | Thank you for this comment. The reference list has now been updated and full access information has been provided for all references. |
| E11: Please correct the typo in reference 20. | The reference has been corrected. Thank you for noting this. |
| E12: Please add the full author names to reference 33. | The full author names have been added. |
| E13: Please remove all publisher names (e.g., "Elsevier") and iterations of "[Internet]" from the reference list. | Publisher names and iterations of Internet have been removed where appropriate. |
| E14: Please adapt the attached CONSORT checklist so that individual items are referred to by section (e.g., "Methods") and paragraph number rather than by page or line numbers, as the latter generally change in the event of publication. Please refer to this in the text as "See S1_CONSORT_Checklist" or similar, and amend the attachment label to match. | We have updated the CONSORT Checklist as indicated, amended the file name, and edited the reference to the supplementary file in the main body (Methods – Design). |

# *** Reviewer #1:

This manuscript is well-written and easy to follow. I just have a few, relatively minor, comments listed below:

| R1C1: Looking at Table 2, patients randomised to the Control group appear to me as if being slightly less healthy that those randomised to the intervention group. For example, 43% vs 31% were identified as mildly frail or worse in the control and intervention groups respectively. I also note some differences in ISAR score and nutritional status. I would like the authors to consider describing these differences in the text as well as consider running sensitivity analyses that adjust for these baseline imbalances. I realise that a non-parametric test was used to assess differences in the primary outcome (length of ED stay); however, as a sensitivity analysis one could consider using a linear model which allows covariates. | We thank the Reviewer for this comment. We calculated the statistical significance of baseline differences, as requested by another Reviewer (see R2C9). While the p-values are not reported in the paper in line with the CONSORT recommendations, we found that there were no statistically significant differences between intervention and control group in terms of ISAR score, frailty or nutrition status. Based on this, it was felt that running sensitivity analyses controlling for these measures would be not necessary. We have included a description of these results at p.17:  “*In terms of baseline differences between the two groups (see Table 2), we observed that patients in the control group were slightly older than those in the intervention group and more likely to be in triage category 3; whereas participants in the intervention group reported more pain or discomfort than those in the control group.*  As we observed significant differences in triage category (a measure of severity of conditions) and age (control group older than intervention group), we decided to run sensitivity analyses on both ED length of stay and rates of hospital admissions controlling for this measure as well as age (as control group was older than intervention. As shown below, we found that the differences between the two groups remained significant even after controlling for triage category and age. We have included the results of this analysis in the Results section p.19:  “*To account for the potential influence of clinical severity,* *and considering the baseline differences in triage category and age, we conducted sensitivity analyses controlling for triage category and age both for ED length of stay (using linear regression) and for rates of hospital admissions (logistic regression); in both cases, the differences between intervention and control group were statistically significant even when considering potential differences in clinical conditions*”  **ED length of stay (linear regression)**  *Unadjusted model*:   - Significant effect of Arm, b = -6.61, p < 0.001. - Overall model: F(1,351) = 14.30, p = 0.0002, R-squared = 0.036.   *Model adjusting for triage category and age*:   - Significant effect of Arm, b = -6.58, p < 0.001. - Significant effect of Triage category (Ref. cat 2): category 3, b = -6.53, p = 0.024; category 4, b = -12.45, p = 0.005. - Significant effect of age: b = 0.27, p = 0.03 - Overall model: F(4,348) = 7.47, p < 0.001, R-squared = 0.068.   **Rates of hospital admissions (logistic regression)**  *Unadjusted model:*   - Significant effect of Arm, OR = 0.18, p < 0.001. - Overall model: Likelihood ratio Chi2(1) = 52.06, p < 0.001.   *Model adjusting for triage category and age*:   - Significant effect of Arm, OR = 0.19, p < 0.001. - No significant effect of triage category only for category 3 compared to cat 2 OR = 1.13, p = 0.76. No effect for category 4 OR 0.27, p = 0.09. - Overall model: Likelihood ratio Chi2(4) = 75.73, p < 0.001. |
| --- | --- |
| R1C2: Is it possible for the authors to add a table describing the intervention as delivered such as the types of activities performed and/or the time spent with the patient? | We have now included a table which provides detailed information about the intervention as a supplementary file (S2_InterventionCharacteristics). Reference to the supplementary table is included in Intervention section, p.11 |
| R1C3: Please add the fact that the study was conducted in a single centre as one of the limitations (lack of generalisability). | We have added this information to the Limitations section, p.25 |
| R1C4: Assuming that the same HSCP staff interacted with patient randomised to both arms, I would like to understand whether contamination (i.e. HSCP staff doing more under usual care than they would normally) occurred and what was put in place to prevent it. | HSCP services to the control group were not provided by the dedicated HSCP team in the ED department. This group accessed HSCP services in the same manner as was in place previous to the implementation of the trial. This has been clarified in the methods section (p.12): “*services to the control group were provided by HSCPs that were not part of the dedicated HSCP team in the ED*” |
| R1C5: A number of patients were excluded from the analysis of ED and hospital admissions at 30 days and 6 months due to death or lost-to-FU. Instead of excluding these patients, one could consider an alternative approach that take exposure/censoring into account. Possible methods include calculating rates (e.g. rates of admission) while adjusting for exposure or using a time-to-event analysis censored for death or loss-to-FU. One would need to consider how to handle death e.g. as a competing risk. I would encourage the authors to consider some of these options as a sensitivity analysis. | We thank the Reviewer for this suggestion. However, at 30 days, we had data for all participants on all objective measures, including ED re-visits and unscheduled hospital admissions, and at 6-months, only 7 patients were excluded from the analysis. Hence, given the small number of patients excluded, we did not consider further time to event analysis. |
| R1C6: Please consider adding the pre-specified analysis plan in the appendix. | We have included a reference to our trial protocol, which includes pre-specified analysis plan, in the Data analysis section, p.13 |

# *** Reviewer #2:

Randomised controlled trial of the impact of a Health and Social Care Professional team in the emergency department on the quality, safety, and clinical effectiveness of care for older adults.

Thank you for the opportunity to review this interesting paper.

The paper provides results from a randomized trial aiming to assess the quality, safety, and clinical effectiveness of an intervention based on an early assessment and intervention by a dedicated Health and Social Care Professionals (HSCPs) comprising a senior medical social worker, senior occupational therapist and senior physiotherapist, to promote a safe and timely discharge for older adults in ED.

The study is very nice and explore a topic which is important for older adults.

Older adults in the Emergency Department are at risk of multiple adverse outcomes. The ED is an unsuitable setting for their care, given the scarcity of staff, the long boarding time, the impossibility of ensuring adequate nursing care (i.e. mobilisation, hydration).

The ED Lenght of stay is associated with a higher risk of delirium, an increased rate of in-hospital admissions and in-hospital lkenght of stay , and an increased mortality rate, also after discharge. Treducing A reduction in the length of stay in ED contributes to reduce the overcrowding , which is one of the main factors that affect the quality of care in this setting.

The authors found a significative reduction in length of stay and in the rate of in-hospital admissions. The intervention is effective to improve function, mobility and self care after 30 days and six months after discharge and the satisfaction for the ED admission is higher in intervention group than in control group.

The aim of the study is clear, the title is relevant and informative, the references are recent and relevant. The authors published recently a review about the topic. The paper is well written and clear.

The introduction explains clearly what is already know about this topic and the introduction provide a strong rationale for the study. The research question is clearly outlined and the study is innovative due to the lack of RCT in this topic.

Despite this, I have some comments about the paper.

| **The main outcome of the study is the length of stay in Emergency Department.**  R2C1: Please, specify what did the authors mean for length of stay: time between arrival and discharge? Time between clinical examination and discharge? Time between arrival at triage and clinical examination? | Thank you for the kind and constructive feedback.  We have now clarified in the outcome section, p.12, that ED length of stay represented the “*duration of stay in the ED in hours from the time of arrival in the ED to the time of discharge*” |
| --- | --- |
| R2C2: The baseline assessment is comprehensive and in the paper is not specified the time needed to complete it. The time spent for the evaluation is included in the length of stay? | Yes, the overall ED length of stay is inclusive of baseline assessment and HSCP assessment and evaluation for participants randomised to the intervention group. While we did not quantify precisely the time for the baseline assessment, the average time taken to complete baseline assessment was approximately 30 minutes.  We have included this information in the Baseline Data collection section (p.10) |
| R2C3: ‘…’Patients who presented with a MTS score of very urgent and were categorised as chest pain, abdominal pain, shortness of breath or headache were invited to take part following consultation with the EM doctor and completion of a medical and diagnostic work-up.’’ What is the effect of the intervention on the length of stay in patients who are enrolled after the completion of the medical and diagnostic investigations? | We thank the Reviewer for this observation. We have now clarified in the Participants section (p.9) that “*Patients who presented with a MTS score of 2 (very urgent) or any of the MTS categories not listed above were invited to take part following consultation with the EM doctor and completion of diagnostic work-up, to determine suitability and indication for HSCP assessment e.g. MTS score 2 presenting with falls, life threatening condition/injury out-ruled and medically stable*”  While we do not have a precise estimate of the effect suggested here, we conducted an analysis of ED length of stay by triage category, as a proxy way to identify patients who could have seen the EM doctor before seeing the HSCP team (MTS category 2). Using a Kruskal-Wallis of between-subjects differences, we found a significant increase in median ED length of stay with increasing urgency of triage category, chi2(2) = 11.02, p = .004:   - MTS 4: Median n. hours = 4.35, IQR = 4.45 - MTS 3: median = 9.40, IQR = 13.14 - MTS 2: median = 12.09, IQR = 24.99   However, as noted in response to comment R2C5, we run sensitivity analyses on both ED length of stay and number of admissions from the ED controlling for patient age and triage category, and found the differences between intervention and control group did not change after controlling for triage category and age. This has been reported at p.19 of the Results section. |
| R2C4: It is well established that the reasons for a prolonged length of stay in ED are several: waiting time for diagnostic exams, waiting for consultants, waiting time for boarding patients…  *… interventions prescribed by the HSCP … included prescription of mobility aids and enabling ADL equipment, provision of home exercise programmes, education of self-management strategies and onward referral to alternative care pathways, as deemed appropriate.*  Please, provide more informations about the intervention and explain how the intervention can reduce the time spent in ED. | We have now included a table which provides detailed information about the intervention as a supplementary file (S2_InterventionCharacteristics). Reference to the supplementary table is included in Intervention section, p.11 |
| R2C5  **Patients with different clinical conditions were enrolled in the study.**  Patients with chest pain, abdominal pain, dyspnea and headache have different needs in ED than patients with minor complaints. For example, a patient with chest pain often requires several blood exams, taking more time than other complaints. So, patients who are admitted in ED with chest pain or abdominal pain or shortness of breath are expected to have to have a much longer waiting time than others.  ‘*’Participants in the intervention group (n=176) experienced a significantly shorter ED stay when compared to the control group (n=177) (6.4 vs. 12.1 median hours, p < 0.001) and had a significantly lower rate of hospital admissions from the ED (19.3% vs. 55.9%, p < 0.001). ‘’*  Is it possible that this difference is related to the reason for admission in ED? How the intervention can reduce the rate of in-hospital admission? The decision to admit a patient in hospital is related to the clinical condition and, sometimes, to a social problem. The decision to admit or discharge a patient is a medical disposition and I’m not sure that the team can influence the decision to discharge a patient. It is not clear how the intervention can influence a clinical decision.  The difference found in the rate of hospital admission is very high, but probably the efficacy of the intervention in the rate of admission is different for patient whit minor complaints than in other.  The reduction in length of stay and in the rate of hospital admission are probably different among patients admitted with 2 or 4-5 MTS score. | We thank the Reviewer for this comment, which is indeed important.  To address this point, we ran a sensitivity analysis both for ED length of stay and rates of admission while controlling for triage category (MTS) and age. We felt that this could give us a useful and meaningful proxy of clinical condition while maintaining some analytical power which we could have lost by comparing multiple complaint categories.  For this analysis, we conducted two separate regressions, one for each outcome. We found that the significant differences were not affected by triage category, as shown below:  **ED length of stay (linear regression)**  *Unadjusted model*:   - Significant effect of Arm, b = -6.61, p < 0.001. - Overall model: F(1,351) = 14.30, p = 0.0002, R-squared = 0.036.   *Model adjusting for triage category and age*:   - Significant effect of Arm, b = -6.58, p < 0.001. - Significant effect of Triage category (Ref. cat 2): category 3, b = -6.53, p = 0.024; category 4, b = -12.45, p = 0.005. - Significant effect of age: b = 0.27, p = 0.03 - Overall model: F(4,348) = 7.47, p < 0.001, R-squared = 0.068.   **Rates of hospital admissions (logistic regression)**  *Unadjusted model*:   - Significant effect of Arm, OR = 0.18, p < 0.001. - Overall model: Likelihood ratio Chi2(1) = 52.06, p < 0.001.   *Model adjusting for triage category and age*:   - Significant effect of Arm, OR = 0.19, p < 0.001. - No significant effect of triage category only for category 3 compared to cat 2 OR = 1.13, p = 0.76. No effect for category 4 OR 0.27, p = 0.09. - Overall model: Likelihood ratio Chi2(4) = 75.73, p < 0.001.   We feel that these analyses further support our findings.  We have now included this information in the Results section p.19:  “*To account for the potential influence of clinical severity, and considering the baseline differences in triage category and age, we conducted sensitivity analyses controlling for triage category and age both for ED length of stay (using linear regression) and for rates of hospital admissions (logistic regression); in both cases, the differences between intervention and control group were statistically significant even when considering potential differences in clinical conditions*” |
| R2C6: In the limits section the authors stated  *We focused on a specific cohort of ‘lower urgency’ patients with a range of index complaints that were amenable to HSCP intervention.*  However, the inclusion criteria state that patients with an acuity level 2 were enrolled after clinical examination. I’m not convinced that chest pain or abdominal pain can be considered fully amenable to HSCP intervention. | We thank the Reviewer for this observation. We agree that the original wording was confusing. We have now clarified in the Participants section (p.9) and in Table 2 the following:  “*Patients who presented with a MTS score of 2 (very urgent) or any of the MTS categories not listed above were invited to take part following consultation with the EM doctor and completion of diagnostic work-up, to determine suitability and indication for HSCP assessment e.g. MTS score 2 presenting with falls, life threatening condition/injury out-ruled and medically stable*” |
| **inclusion and exclusion criteria**  R2C7: The inclusion and exclusion criteria are not stated clearly. In the method section *Prospective patients who had a Manchester Triage System score (MTS) of urgent, standard or non-urgent and who were categorized at triage with limb problems, falls, unwell adult, back pain, urinary problems or ear/facial problems were invited to take part.*  But in table 2 there are listed 65 patients admitted for other conditions; major trauma, gastrointestinal bleeding, collapsed adult; gastrointestinal bleeds, head injury... Please, specify the inclusion and exclusion criteria. | We have now clarified in the Participant section and in Table 1 the process and criteria through which patients were included or excluded from the study.  The list of 3-5 presented in Table 1 refers to the MTS categories that were proactively enrolled by the team prior to EM work-up out of the 54 Manchester triage categories. All other categories in results table were recruited post EM work-up and suitability determined following discussion with Doc.  We have clarified in Table 2 that “*Index complaints are presented here from most to least common, independent of MTS category*” |
| R2C8: Medically unstable: please, specify what this means (ie blood pressure under 90 mmHg, heart rate, news score…). Change with ‘’immediate’’ if the authors mean ''1'' code in MTS scale, or state if it refers to patients unstable also after the clinical examination and treatment. Patients admitted with very urgent condition are clinically unstable, but they were enrolled after medical examination. Please, clarify these inclusion and exclusion criteria. | We acknowledge that the terms “medically unstable” or “medically stable” presented in Table 1 were vague.  We have now clarified replaced these with the corresponding MTS scores in Table 1. Medically stable has been replaced with “MTS 3-5”, while medically unstable has been replaced with MTS 1-2.  For MTS 1-2, we have clarified in the table notes that “*MTS score 1-2 only recruited after Emergency Medicine diagnostic work-up and suitability for HSCP assessment determined*”  We have also updated the text under participants (pp.8-9) to better clarify the process through which patients with different MTS scores were recruited in the trial. |
| **Minor comments:**  R2C9: Please, provide a p-value for characteristics of the sample showed in table 1 | We thank the Reviewer for this suggestion. We have not reported the p-values in table 2 in line with CONSORT recommendations (<http://www.consort-statement.org/checklists/view/32--consort-2010/510-baseline-data>). According to these recommendations, looking at differences based on p-values might mean some important variables may be omitted (as there was no evidence of a significant difference) and the inclusion of irrelevant covariates (not as important). It has been proposed that one adjusts for known important prognostic variables, and specify these in the protocol, as it was done for this study. Based on this recommendation, the research team is of the opinion that p-values should not be presented in the manuscript. |
| R2C10: Table 2: A category is missed in table 1 for MTS score (2:very urgent-3:urgent-4standard?). Patient non urgent? | We have clarified in the Results section p.17:  “*Patients included in the trial presented a MTS score ranging from 2 “Very urgent” to 4 “standard”, while no patients with a score of 1 (non-urgent) or 5 (non-urgent) were part of the sample*” |
| R2C11: It could be interesting to assess the delta in the value of Barthel index and EuroQol in the same patient. An improvement after discharge can be reasonable due to the resolution of the acute clinical condition. | We thank the Reviewer for this suggestion. We note that all analyses on follow-up function and quality of life controlled for performance at baseline. However, we calculated delta scores and conducted some sensitivity analyses, as indicated below.  We have reported the results of these comparisons in the manuscript at p.21 and 22.  **Barthel**  As suggested by the Reviewer, we calculated the delta of Barthel at the two follow-ups as follows:   - T1DeltaBarthel, as the difference between Barthel at 30 days and at baseline - T2DeltaBarthel, as the difference between 6 months and 30 days.   For T1DeltaBarthel (30 days), we found that participants in the intervention group had a significant improvement from baseline to 30 days (M = 0.21, SD = 2.91) compared to those in the control group (M = -0.82, SD = 3.14); diff = -1.02, t(321) = -3.04, p = 0.003, Cohen’s d = 0.34.  We regressed the delta measure by arm, controlling age, ISAR score and triage category (as shown in the STATA output below), and found that the difference in function between intervention and control group was significant (b = 0.96, p = .005).  For T2DeltaBarthel (6 months), we found no significant difference between the two groups (intervention, M = -0.03, SD = 2.32; control, M = 0.08, SD = 2.58; t(282) = 0.39, p =0.69. This absence of significant differences was maintained when controlling for age, ISAR and triage category (see STATA output below).  **EuroQOL**  Similarly, we calculated the deltas for all subdomains of quality of life.  At 30 days, we found no statistically significant differences between intervention and control group for any of the subdimension of the EuroQOL:   - T1DeltaMobility: t(327) = 1.66, p = 0.09 - T1DeltaSelfcare: t(327) = 1.86, p = 0.06 - T1DeltaUsualActivities: t(327) = 0.78, p = 0.43 - T1DeltaDiscomfort: t(327) = 1.26, p = 0.21 - T1DeltaAnxiety: t(325) = 0.37, p = 0.71   The same was observed at 6 months:   - T2DeltaMobility: t(291) = 0.12, p = 0.91 - T1DeltaSelfcare: t(291) = 1.39, p = 0.16 - T1DeltaUsualActivities: t(291) = 0.28, p = 0.77 - T1DeltaDiscomfort: t(291) = -0.07, p = 0.94 - T1DeltaAnxiety: t(291) = 0.85, p = 0.39   This absence of differences remained for all measures both at 30 days and follow-up when controlling for age, ISAR and triage category (data not reported here).  We have reported the results of these comparisons |

T1DeltaBarthel by Arm, controlling for age, ISAR and triage category

T2DeltaBarthel by Arm, controlling for age, Isar and triage category

| R2C12: In the sample size it was estimated a 40% decrease in ED length of stay in the intervention group. The authors hypothesized a high reduction in the length of stay, despite previous studies are discordant. For example, Arendts and colleagues reported no significant differences in terms of hospital length of stay after admission from the ED between ED-based CCT assessment of older patients as compared to routine medical assessment (median length of stay: 88 hours in intervention vs. 87 in control, IRR = 0.97, p = 0.32). | Thank you for this comment.  We based our sample calculation on the findings by Taylor and colleagues (now reference 15). We have now clarified this point in the sample size section, p14. |
| --- | --- |
| R2C13: How did the researchers calculate the MMSE score? | Apologies for the inclusion of the MMSE in the table. The method of ascertaining capacity to consent has been clarified in Table 2: *“In cases where there was a clinical concern regarding capacity to consent, the 4AT tool was used to screen for cognitive impairment and in participants where there was evidence of moderate-profound impairment, the patient’s nominated contact person was contacted for consent*” |
| R2C14: Follow-up: *The research nurse collected data on objective measures (e.g., ED length of stay, admission rates, ED re-visits, unscheduled hospital admissions) using the hospital routine databases, …* Is it possible that ED visits or hospitalizations in other hospital than the hospital of the study have been lost? Is the hospital routine database inclusive of other hospitals? | We have described the hospital where the study took place in the context of the larger hospital group. See design section p.8.  We have also clarified the data available at follow-up through the hospital database (see data collection, p.13): “*A hospital attendance or admission at any of the three smaller local hospitals in this hospital group would also be captured on this database*”. |
| R2C15: Did the telephone call for the follow-up included a reminder of indications provided at discharge? | No, the follow-up calls were aimed only at collecting follow-up data. This has been clarified in the data collection section (p.13): “*No reminder of indications provided at discharge was given during the follow-up phone calls*” |
| R2C16: Please, describe the setting (ie number of patients admitted per year, rate of patients whit low severity at triage per year) and the place where the evaluations were performed (a dedicated room?) | We have now included this information in the Design section, p. 8:  “*The university teaching hospital sits within a larger hospital group with six hospital sites but all function as a single hospital system caring for a substantially rural population of approx. 385,000. The teaching hospital is the only hospital in the group that has a full 24/7/365 emergency care and critical care service and has 455 inpatient beds*.”  We have also included at p.12 that “*The HSCP team had a dedicated cubicle in the ED where they performed assessment and intervention, as appropriate. However, a number of assessments were completed trolley-side after individual risk assessment*.” |

# *** Reviewer #3:

General comments:

This is an interesting, important and timely study on improving post-ED outcomes for older adults seen in the ED utilizing a multidisciplinary HSCP team comprised of a social worker, occupational therapist and physiotherapist. The impressive reductions in ED length of stay and hospital admission rates, as well as improvements in 30-day function, mobility and self care generate enthusiasm for this potentially relatively uncomplicated intervention.

| R3C1: However, one important aspect that limits the ability to apply and generalize the results to other settings is some description of what exactly the scope of the HSCP team was on patient care. Was it an assessment only with communication of findings to the medical team for them to determine treatment and follow-up care, an in-ED treatment intervention, an intervention including self-practice skills for home, a transition of care to an outpatient provider, or something else? The discussion suggests that transition of care did not occur, but more explicit description in the main methods and results would be important. If it was a combination, what percentage of cases fell into the various categories or multiple categories? How was a tailored approach determined if used? How long did the assessment and intervention take, and how many patients was the team actually able to intervene on in a day? Understanding the level of expertise and resources required will help inform how practical or translatable the content of the intervention is to other EDs. | We thank the Reviewer for these points.  We have now included a table which provides detailed information about the intervention as a supplementary file (S2_InterventionCharacteristics). Reference to the supplementary table is included in Intervention section, p.11 |
| --- | --- |
| R3C2  Additional specific comments below for clarification of some of the sections:  Abstract:  R3C2: Participants: It would be helpful to include a couple words describing the inclusion criteria and/or whether this was a convenience sample, to give the reader a better sense from the abstract who the intervention was applied to. E.g. patients presenting between certain hours, and/or the overarching theme of the index complaints (e.g. illness or injury causing impairment of baseline functional status). | We have now clarified in the Abstract the following:  “*This single site randomised controlled trial included a sample of 353 patients aged ≥65 years who presented with low urgency complaints to the ED a university hospital in the Mid-West region of Ireland, during HSCP operational hours*” |
| Introduction:  R3C3: Page 6: The second sentence could be re-worded for clarity and grammar (e.g. incremental *increases* in ED attendance) | We have reworded the sentence to the following (see p.6):  “*A growing ageing population and a higher number of individuals with multi-morbidity are among the main demographic drivers of increases in ED attendances* ” |
| R3C4: Page 7: First sentence of the second paragraph has multiple typographical and wording errors that make it confusing as to what the target outcome measures are. Please clarify to make it clear what the target outcomes are. | We have amended the wording of the sentence to read as follows:  “*The implementation of quality improvement strategies that can help to streamline patient flow in the ED has been shown to enhance quality of care [6]; these strategies are especially important for older adults, whose health outcomes are negatively impacted by frequent ED attendances [7,8] and ED crowding*” |
| R3C5: Page 8: Participants and recruitment: As in the abstract, it would be helpful to give a brief description of the theme of the inclusion criteria to better understand the target population (e.g. older adults whose illness or injury cause impairment of baseline functional status). | We have expanded on Table 1 to add the following at p.8:  “*Patients were eligible for participation in the study if they were aged ≥65 years, medically stable, able to provide written consent, and presented to the ED during HSCP operational hours with one of the index complaints included in Table 1*” |
| R3C6: For the very urgent cases, would be helpful to specify what criteria were used after the work-up to deem them able to be invited (e.g. life-threatening causes ruled out, or likely to be discharged). | We have now clarified in the Participants section (p.9) that “*Patients who presented with a MTS score of 2 (very urgent) or any of the MTS categories not listed above were invited to take part following consultation with the EM doctor and completion of diagnostic work-up, to determine suitability and indication for HSCP assessment e.g. MTS score 2 presenting with falls, life threatening condition/injury out-ruled and medically stable*” |
| R3C7: Page 10: Baseline data collection: For falls and hospitalizations, do you mean incidence (which is a proportion and usual population-based), or *number* of times for the individual? This seems like it should be "number"; if so, please re-word. | Thank you for pointing this out. We have corrected the wording to indicate that these measures referred to the following (see p.11)  “*whether the person had experienced any falls in the three months prior to the ED visit; whether the person have been hospitalised in the six months prior to the ED visit”* |
| R3C8: Page 11: Unclear what is meant by the term "bleeped". Please rephrase for clarity. | We have amended the term to “*requested*”(see p.12) |
