# Supplementary material for: Impact of assessment and intervention by a health and social care professional team in the emergency department on the quality, safety, and clinical effectiveness of care for older adults: A randomised controlled trial
Source: PLoS Med. 2021 Jul 28;18(7):e1003711. doi: 10.1371/journal.pmed.1003711 (PMC8318294; doi:10.1371/journal.pmed.1003711)
Supplement: Attachment — Submitted filename: PMEDICINE-D-20-05367R2_ResponseToReviewers.docx [file pmed.1003711.s006.docx]

**Subject**: Submission of Revised Research Article (R2) titled “*Impact of assessment and intervention by a Health and Social Care Professional team in the emergency department on the quality, safety, and clinical effectiveness of care for older adults: a randomised controlled trial*”.

Response to Editor and Reviewers

# **Editors**

**E1: Please make that "data ... are" in your data statement (submission form).**

**Response** – Wording amended in the submission form.

**E2: You may wish to note the element of assessment (by the team) in your title.**

**Response** – We have amended the title to include “assessment and intervention”, so to reflect the two main tasks of the HSCP team. The title now reads: “*Impact of assessment and intervention by a Health and Social Care Professional team in the emergency department on the quality, safety, and clinical effectiveness of care for older adults: a randomised controlled trial.*”

The title has been amended in any document submitted, and in the submission form.

**E3: Please substitute a colon for the semicolon in your title.**

**Response** – Amended as required.

**E4: In the Methods section, please refer to figure 1 as "Participant flowchart" or similar rather than "CONSORT flowchart".**

**Response** – This has been amended as required (see p.8). The figure caption has also been amended to state “*Participant Flowchart, based on the CONSORT 2010 diagram*”

**E5: Please remove the information on ethics approval from the end of the main text (this belongs in the Methods section) and the information on transparency and dissemination.**

**Response** – Both statements have been removed. Information on ethics approval has been moved to the Methods section, under Design, p.8.

**E5: Please use the abbreviation "PLoS ONE" in your reference list.**

**Response** – We identified two references relevant to this comment, respectively #4 and #16. Both references have been amended as required.

# ***** Reviewer #1**

**Most of my comments have been adequately addressed; however, I have one remaining issue which relates to the adjustment for baseline imbalances. The authors state that it was not necessary to adjust for differences in ISAR score, frailty or nutrition status given that tests comparing the two arms at baseline were not significant. I believe that using significance tests to decide whether or not to adjust for baseline differences is potentially misleading due to the following reasons:**

**1) p-values measure the degree of consistency between the null hypothesis and the observed data. In the case of a randomised trial, we know that the null hypothesis is in fact true (i.e. that there is no true difference between the two randomised arms) and that any observed difference is due to chance.**

**2) One may not have the appropriate power to test for baseline differences For these reasons, I believe it is important to decide whether or not to adjust, not based on tests of significance, but on observed differences. To quantify the differences, one option is to use standardised differences which do not depend on the sample size.**

**I would therefore strongly suggest adding the ISAR score, the frailty status and the nutrition status to the adjusted model already containing the triage category.**

**Response**: We thank the Reviewer for the valuable comment. As requested, we re-ran the sensitivity analyses for ED length of stay (linear regression, Sensitivity Analysis Table 1 below) and number of admissions (logistic regression, Sensitivity Analysis Table 2 below), controlling for triage category, patient age, ISAR score, nutritional status, and frailty status. The effect of the intervention remained significant in the adjusted models, for both outcomes, as outlined in the Sensitivity Analysis Tables below.

In order to make these supplemental analyses accessible to the Reader, we have included them into a new supplementary file S3_SensitivityAnalyses, and this has been signposted in the Results section, which has been amended as follows (p.19): “*To account for the potential influence of clinical severity, and* *considering potential baseline differences in triage category, age and health status, we conducted sensitivity analyses controlling for triage category, age, ISAR score, nutritional status, and clinical frailty score, both for ED length of stay (using linear regression) and for rates of hospital admissions (logistic regression); these can be seen in the S3_SensitivityAnalyses tables. For both measures, the differences between intervention and control group were statistically significant even when considering potential differences in clinical conditions*”.

| **Sensitivity Analysis Table 1 \| ED length of stay – Linear Regression** | | | | | | |
| --- | --- | --- | --- | --- | --- | --- |
|  | **Model 1** | | | **Model 2** | | |
| **Measure** | **b** | **p-value** | **95% CI** | **b** | **p-value** | **95% CI** |
| Arm (Ref: Control) | -6.61 | < 0.001 | -10.04: -3.17 | -5.72 | 0.001 | -9.12: -2.33 |
| Triage category (Ref: MTS 2) |  |  |  |  |  |  |
| MTS 3 |  |  |  | -6.66 | 0.019 | -12.21 ; -1.12 |
| MTS 4 |  |  |  | -11.85 | 0.007 | -20.43 : -3.28 |
| Age |  |  |  | 0.15 | 0.23 | -0.09 : 0.41 |
| ISAR score (Ref: <2) |  |  |  |  |  |  |
| >=2 |  |  |  | 1.75 | 0.46 | -2.97 : 6.48 |
| Nutritional status (Ref: 1. Malnourished) |  |  |  |  |  |  |
| At risk of malnutrition |  |  |  | 5.38 | 0.13 | -1.54 : 12.31 |
| Normal status |  |  |  | -0.89 | 0.79 | -7.73 : 5.93 |
| Clinical Frailty Score |  |  |  | 1.27 | 0.09 | -0.23 : 2.78 |
| F | 14.30 | < 0.001 |  | 6.40 | < 0.001 |  |
| Adjusted R^2^ | 0.03 |  |  | 0.11 |  |  |
| **Notes**. b = unstandardised regression coefficient, CI = Confidence Interval, ED= Emergency Department, F = Anova F-ratio, R^2^ = coefficient of determination, Ref = Reference category. | | | | | | |

| **Sensitivity Analysis Table 2 \| Hospital admissions – Logistic Regression** | | | | | | |
| --- | --- | --- | --- | --- | --- | --- |
|  | **Model 1** | | | **Model 2** | | |
| **Measure** | **OR** | **p-value** | **95% CI** | **OR** | **p-value** | **95% CI** |
| Arm (Ref: Control) | 0.18 | < 0.001 | 0.12 : 0.31 | 0.21 | < 0.001 | 0.12: 0.34 |
| Triage category (Ref: MTS 2) |  |  |  |  |  |  |
| MTS 3 |  |  |  | 1.13 | 0.78 | 0.48 : 2.64 |
| MTS 4 |  |  |  | 0.32 | 0.15 | 0.06 : 1.53 |
| Age |  |  |  | 1.03 | 0.16 | 0.99 : 1.07 |
| ISAR score (Ref: <2) |  |  |  |  |  |  |
| >=2 |  |  |  | 1.61 | 0.21 | 0.77 : 3.35 |
| Nutritional status (Ref: 1. Malnourished) |  |  |  |  |  |  |
| At risk of malnutrition |  |  |  | 1.37 | 0.52 | 0.52 : 3.63 |
| Normal status |  |  |  | 0.58 | 0.26 | 0.23 : 1.51 |
| Clinical Frailty Score |  |  |  | 1.16 | 0.18 | 0.93 : 1.51 |
| Likelihood ratio chi^2^ | 52.06 | < 0.001 |  | 86.31 | < 0.001 |  |
| Pseudo R^2^ | 0.11 |  |  | 0.18 |  |  |
| **Notes**. CI = Confidence Interval, ED= Emergency Department, OR = Odds Ratio, R^2^ = coefficient of determination, Ref = Reference category. | | | | | | |

# ***** Reviewer #2**

**Thank you for submitting the revised version of the manuscript.**

**I thank the authors for taking into account all comments. The answers given are satisfactory. In particular, I appreciate the sensitivity analyses added, which reinforce the results. The description of the intervention characteristics is clear and detailed.**

**I have no more comments.**

**Response**: We thank the Reviewer for the positive feedback.

# ***** Reviewer #3**

**The revised manuscript is much clearer and addresses previous concerns.**

**Response**: We thank the Reviewer for the positive feedback.
