# Supplementary material for: Impact of assessment and intervention by a health and social care professional team in the emergency department on the quality, safety, and clinical effectiveness of care for older adults: A randomised controlled trial
Source: PLoS Med. 2021 Jul 28;18(7):e1003711. doi: 10.1371/journal.pmed.1003711 (PMC8318294; doi:10.1371/journal.pmed.1003711)
Supplement: Attachment — Submitted filename: PMEDICINE-D-20-05367R3_ResponseToReviewer.docx [file pmed.1003711.s007.docx]

**Subject**: Submission of Revised Research Article (R3) titled “*Impact of assessment and intervention by a Health and Social Care Professional team in the emergency department on the quality, safety, and clinical effectiveness of care for older adults: a randomised controlled trial*”.

Response to Editor

**E1: Please quote aggregate demographic details for study participants in the abstract, i.e., age and sex from the rightmost column of table 2.**

**Response**: We have now added aggregate demographic details to the abstract as follows (p.3):

*This single site randomised controlled trial included a sample of 353 patients aged ≥65 years* *(mean age=79.6, SD=7.01; 59.2% female)*

**E2: Where appropriate, e.g., in the abstract, please substitute "sex" for "gender".**

**Response**: We have checked the abstract and rest of the manuscript and confirm that no instances of “sex” are present.

**E3: In the author summary, please use the active voice (e.g., "We aimed ...) in at least one point.**

**Response**: We have now included the active voice for two points, as follows (p.5):

*In this randomised controlled trial, we evaluated the impact of an ED-based HSCP team dedicated to older patients on ED length.*

*We observed no differences in the numbers of patients re-presenting to the ED at 30 days or 6 months.*

**Reviewer #1:**

**The authors have adequately addressed my last comment. I have no further comment.**

**Response –** We thank the Reviewer for the positive feedback.
